# Integrated Transcriptome and Metabolome Analysis of Salinity Tolerance in Response to Foliar Application of β-Alanine in Cotton Seedlings

**DOI:** 10.3390/genes14091825

**Published:** 2023-09-20

**Authors:** Wei Ren, Li Chen

**Affiliations:** 1 State Key Laboratory of Desert and Oasis Ecology, Xinjiang Institute of Ecology and Geography, Chinese Academy of Sciences, Urumqi 830011, China; chenli1@ms.xjb.ac.cn; 2 Fukang Station of Desert Ecology, Chinese Academy of Sciences, Fukang 831505, China

**Keywords:** salt tolerance, upland cotton, differentially expressed genes, differentially accumulated metabolites, conjoint analysis

## Abstract

Salinity is amongst the serious abiotic stresses cotton plants face, impairing crop productivity. Foliar application of β-alanine is employed to improve salt tolerance in various crops, but the exact mechanism behind it is not yet completely understood. An advanced line SDS-01 of upland cotton *Gossypium hirsutum* L. was utilized to determine its salt tolerance. Foliar treatment with the β-alanine solution at different concentrations was applied to the seedlings stressed with 0.8% NaCl solution. On the 10th day of treatment, samples were collected for transcriptome and metabolome analyses. β-alanine solution at a concentration of 25 mM was found to be the best treatment with the lowest mortality rate and highest plant height and above-ground biomass under salt stress. Both differentially expressed genes and accumulated metabolites analyses showed improved tolerance of treated seedlings. The photosynthetic efficiency improved in seedlings due to higher expression of photosynthesis-antenna proteins and activation of hormones signal transduction after treatment with β-alanine. Highly expressed transcription factors observed were *MYB*, *HD*-*ZIP*, *ARF*, *MYC*, *EREB*, *DELLA*, *ABF*, *H2A*, *H4*, *WRKY*, and *HK* involved in the positive regulation of salinity tolerance in β-alanine-treated seedlings. Furthermore, compared to the control, the high accumulation of polyamines, coumarins, organic acids, and phenolic compounds in the β-alanine-treated seedlings helped regulate cellular antioxidant (glutathione and L-Cysteine) production. Hence, to improve salt tolerance and productivity in cotton, foliar application of β-alanine at the seedling stage can be a valuable management practice.

## 1. Introduction

Plants are often exposed to various stresses under natural field conditions, negatively impacting their performance. Among them, salinity and drought cause significant losses in crop productivity worldwide [1]. Salinity is the major abiotic stress affecting crop productivity across semi-arid to arid crop-growing areas [2]. The seedling establishment stage is highly prone to salinity due to several adverse changes in biochemistry and physiology that ultimately create different stresses, viz., oxidative, ion-specific, and osmotic stress [1,3]. The delayed seed germination and establishment are caused by reduced water availability, protein structural disorganization, and impaired mobilization of reserves [4].

It is believed that climate change, long-term drought spells, and sea levels rising are the reason for this substantial expansion in salinity-affected areas. The Food and Agriculture Organization (FAO) reports indicated that around 0.424 billion hectares of the soil surface and 0.833 billion hectares of subsoil around the globe have been categorized as salt-affected [5]. Other studies reported that salt-affected areas will increase to around one billion hectares worldwide [6]. Xinjiang, the northwest province of China and a major cotton growing area, has been reported as salt-affected, with approximately one-third of arable land [7]. Its cotton production reached around 2.5 million hectares during the current year [5]. This province contributes to 24% of total cotton production in China (https://ipad.fas.usda.gov, accessed on 26 August 2022), which is now seriously getting affected due to salinity stress [8].

Upland cotton (*Gossypium hirsutum* L.) is moderately salt-tolerant and can easily survive in the saline medium of 7.7 dS/m salt concentration [9] as compared to other important crops [10,11]. Moreover, a wide range of highly adaptable cotton cultivars are available, harboring tolerance against salinity. It is considered that salinity could potentially threaten cotton productivity in the upcoming years [12,13]. The crop’s susceptibility to salinity largely depends on the developmental stage and salt type. Reasonable knowledge about cotton response to salinity and tolerance mechanisms is crucial for designing management practices for better productivity in saline growing medium [14]. Several studies, including whole genome sequencing for various cotton species, helped to understand better the diverse set of alleles/genes activated under stressful conditions that might control various biological and molecular metabolic pathways [15,16,17,18,19,20].

**Figure 1:**
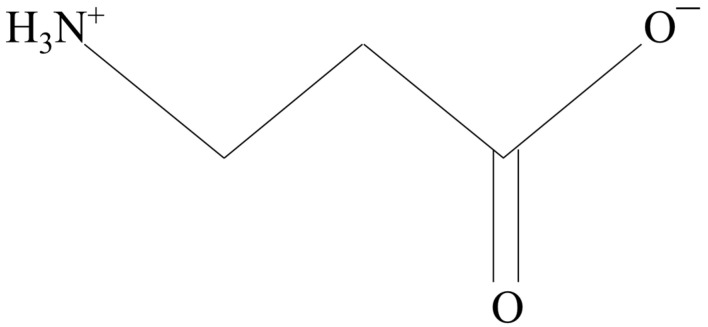
The chemical structure for β-alanine (a non-proteinogenic amino acid) [32].

Numerous approaches have been designed to combat the detrimental effects caused by abiotic stresses, particularly salinity [1,21,22,23,24]. Foliar application of different chemicals to make plants tolerant to stressful (drought and salinity) environments has emerged as an effective practical strategy [25,26,27]. Several plants tend to accumulate various quaternary ammonium compounds (QACs) in response to different abiotic stresses (salinity and drought in particular) [28]. These compounds increase the osmotic pressure across the cytoplasmic membrane without compromising metabolic activities [29]. Similarly, it has been discovered that highly stress-tolerant species of the family Plumbaginaceae accumulate β-alanine to compensate for the adverse effects of salinity (Figure 1). Hence, β-alanine is thought to be an appropriate osmoprotectant against saline hypoxic conditions [30,31], as its accumulation in seedlings or plant parts has been reported to activate different genes and pathways involved in salinity tolerance [32,33].

Various studies revealed that cotton plants are more prone to salinity stress at the seedling stage than in later stages [34,35,36]. In response to salt stress, the reduced metabolic activities of enzymes, viz., ABA, gibberellins, cytokinins, auxins, alkaline invertase, salicylic acid, acidic invertase, and sucrose phosphate synthase, causes deterioration of lint quality in cotton [13,17]. The knowledge of molecular mechanisms related to salinity tolerance can be enhanced by determining the genes expressed in a stressed medium/environment. The discovery of such genes has been reported in earlier studies on salinity tolerance, followed by their utilization in developing new resilient/adaptable cotton varieties. For example, some reported salinity-tolerant genes in cotton are *GhNAC* [37,38], *GhMT3a* [39], *GhRLK* [40], *GhWRKY* [41,42], *GhZFP1* [43], and *GhDREB* [44].

Numerous genome-wide investigations have identified salt-responsive gene families in cotton [16,45,46,47]. The advancement of bioinformatics tools and next-generation sequencing technologies significantly aided in identifying and characterizing relevant differentially expressed genes (DEGs) in cotton plants growing in salt-stressed mediums [41,47]. In recent years, the availability of upland cotton’s updated high-quality reference genome facilitated transcriptome profiling of cotton plants grown under various growth conditions [48,49,50]. Many studies have been conducted to determine the responses of cotton plants against salt stress [34,41,45,46,47], but none have elaborated on the function of β-alanine in upgrading salinity tolerance. Hence, the objective of this study was to evaluate the effect of β-alanine pretreatment on cotton seedling response to salinity stress. The outcome of the current study would have important implications on cotton management and production across salinity-prone arid and semi-arid cotton-growing regions.

## 2. Material and Method

### 2.1. Experiment Design and Sample Collection

The plant material utilized in the current study was an advanced line SDS-01 of upland cotton (*G. hirsutum* L.). The experiment was conducted in the under-protected greenhouse from late December 2021 to early January 2022 at the Nanfan Test Base, Liguo Town, Ledong County, Hainan Province, China. The soil used to grow the seedlings was rich in organic matter, collected from the mountains with gray color and alluvial texture. Following were the physio-chemical properties of the utilized soil, i.e., “pH 8.03, electrical conductivity (EC) (4088 µScm^−1^), Mg^2+^ (0.182 mgg^−1^), Na^+^ (2.473 mgg^−1^), Ca^2+^ (1.776 mgg^−1^), SO4^2−^ (8.49 mgg^−1^), Cl^−^ (0.537 mgg^−1^), K^+^ (0.265 mgg^−1^), and salt content (14.63 mgg^−1^)”. Plastic pots (length: 27 cm; width: 25 cm) used for seedlings growth were filled with 5 Kg of soil each. On the appearance of the 4th leaf, 10 well-growing seedlings were maintained per pot, followed by a spray of β-alanine solution (100 mL) in concentrations of 0 mM (A1), 10 mM (A2), 25 mM (A3), 50 mM (A4), and 100 mM (A5). On the third day after β-alanine spray, the salt stress was induced by adding 1000 mL NaCl (4%) solution, which gave the soil 0.8% salt contents. On the 10th day of stress, the seedlings were investigated in three replicates regarding their mortality rate (%), growth rate (plant height in cm), as well as above-ground biomass (shoot weight in g). During the whole test, the day temperature was maintained at 23–27 °C, and the night temperature was kept at 7–13 °C. Sample collection was carried out from five randomly selected seedlings (above and underground parts) per replicate from each treatment and kept in liquid nitrogen followed by storage at −80 °C.

### 2.2. Transcriptome Profiling

Fifteen independent samples (A1(CK), A2, A3, A4, and A5) in triplicates from ten seedlings were utilized for total RNA extraction. The already-reported protocols were followed for extracting RNA and cDNA library preparation for Illumina sequencing [51]. The Illumina sequencing was accomplished based on the PE150 sequencing strategy.

The clean reads were obtained after processing raw reads, such as by eliminating low-quality bases using threshold Q-value ≤ 20 and poly-N > 10% coupled with adaptors. Furthermore, the obtained cleaned reads were calculated for Q20, Q30, and GC contents. After filtration for clean reads, they were aligned with the reference genome [52] through HISAT2 software v2.2.1 [53]. The Cuffcompare software v2.2.1.0 discovered the new genes [54]. The following databases discovered annotations for all new and original annotated genes: NCBI non-redundant protein sequences (Nr) [55], a manually annotated and reviewed protein sequence database (Swiss-Prot) [56], Protein family (Pfam), Gene Ontology (GO) [57], Trembl [58], Kyoto Encyclopedia of Genes and Genomes (KEGG) [59], and Clusters of Orthologous Groups of Proteins (KOG/COG) [60].

The RSEM software package v1.2.12 [61] was used to calculate the gene expression levels. The fragment/kb of transcript/million mapped reads (FPKM) values were also calculated for each transcription region to estimate variation and abundance in their expression. The threshold criteria for transcript screening were log_2_FC ≥ 2 and FDR < 0.05. The DESeq2 [62] software v1 was used to access the differential expression of RNAs between 2 groups of samples. The database PlantTFDB [63] was utilized to annotate transcription factors (TFs) via iTAK software v18.12.

### 2.3. Metabolome Profiling

#### 2.3.1. Sample Extraction

Sampled seedlings (above and underground parts) have been washed and stored at −80 °C before use for metabolomic analyses. Three samples for each treatment were freeze-dried in liquid nitrogen and then crushed for 2 min at 30 Hz in an MM400 mixer mill (RETSCH, Phoenix, AZ, USA) with the help of zirconia beads. A 100 mg powdered sample was dissolved in 70% methanol (1.2 mL) with the help of a vertex for 30 s. The step was repeated six times, and the samples were stored in the refrigerator at 4 °C overnight. These samples were then centrifuged at 12,000 rpm for 10 min flowed by filtering the extract (SCAA-104, 0.22 μm pore size; ANPEL, Shanghai, China) before UPLC-MS/MS analysis.

The extracted triplicate samples were analyzed in an LC-ESI-MS/MS system (UPLC, Shim-pack UFLC SHIMADZU CBM A system; MS, QTRAP^®^ 4500+ System, SHIMADZU, Kyoto, Japan). For analysis, the UPLC column was Waters ACQUITY UPLC HSS T3 C18 (1.8 µm, 2.1 mm × 100 mm), column temperature of 40 °C, and 0.4 mL min^−1^ flow rate. The injection volume for the samples was 2 μL. The sample system consisted of water (0.1% formic acid) and acetonitrile (0.1% formic acid). The gradient program was set as 95:5 *v*/*v* at 0 min, 5:95 *v*/*v* at 10.0 min, 5:95 *v*/*v* at 11.0 min, 95:5 *v*/*v* at 11.1 min, and 95:5 *v*/*v* at 15.0 min. For ESI-Q TRAP-MS/MS, we used the instrument, settings, conditions, and software reported by Li et al. [64].

#### 2.3.2. Data Analyses

The metabolites were analyzed based on the NMDB database (Norminkoda Biotechnology Co., Ltd., Wuhan, China) and other public databases, as reported by Li et al. [64]. Mass spectral data were processed in Analyst 1.6.3 (Sciex, Framingham, MA, USA). The metabolite quantification was performed in the MRM mode of QQQ MS. Once the metabolite MS data were obtained, we used MultiQuant (3.0.2, AB SCIEX, Concord, ON, Canada) for peak area integration, followed by the determination of the relative metabolite contents using chromatographic peak area.

The unsupervised principal component analysis (PCA), Pearson correlation coefficient (PCC), and hierarchical cluster analysis (HCA) were computed using prcomp and cor functions in R (www.r-project.org (accessed on 3 April 2022)). Orthogonal partial least squares discriminant analysis (OLPS-DA) was performed for the identified metabolites, and the differentially accumulated metabolites (DAMs) were identified between treatment groups. The screening conditions for DAMs identification between the groups were as follows. Fold change ≥ 1.5 and ≤ 0.67 and VIP ≥ 1. The VIP values were extracted from OPLS-DA results done in the R package MetaboAnalyst R (https://github.com/xia-lab/MetaboAnalystR (accessed on 3 April 2022)). The data were log-transformed (log2) and mean-centered before OPLS-DA. A permutation test (200 permutations) was performed to avoid overfitting.

Metabolite annotation was performed in the KEGG compound database (http://www.kegg.jp/kegg/compound/ (accessed on 10 May 2022)). The metabolites that could be annotated were then mapped to the KEGG Pathway database (http://www.kegg.jp/kegg/pathway.html (accessed on 6 April 2022)). The pathways to which the DAMs could be significantly mapped were entered in metabolite sets enrichment analysis (MSEA), followed by determining their significance using the hypergeometric test’s *p*-values.

#### 2.3.3. Conjoint Analysis

The systematic and comprehensive integrated statistical analyses of transcriptome and metabolome data for cotton seedlings treated with the β-alanine solution were conducted to establish the relationships between genes and metabolites. It was performed via a combination of biological functional analyses, correlation analysis, metabolic regulatory pathways, and functional annotation analyses to screen out key genes or metabolic regulatory pathways involved in accumulating various metabolites. Such genes related to growth, development, photosynthesis, salt stress, and enzymatic activities have been selected for this analysis. After normalization, batch data were analyzed via R software in the “cor” package. Pearson’s correlation coefficient R^2^ ≥ 0.8 with *p*-values ≤ 0.05 was used for the correlation analysis and corrected for the Bonferroni multiple tests.

#### 2.3.4. qRT-PCR Experiment

A total of 10 genes were selected from various pathways related to salt stress and used for qRT-PCR expression profiling with the A1 and A3 samples. The total RNA was extracted as explained above. The pure RNA was reverse-transcribed using Transcript United States II one-step gDNA removal and cDNA synthesis supermix (TransGen Biotech Co., Ltd., Beijing, China) according to the manufacturer’s instructions. Primer 5 software was used to design gene-specific primers (Table S1). qRT-PCR assays were performed in triplicate on the Bio-Rad 7500 fast fluorescence quantitative PCR platform with TransStart^®^ top green qPCR supermix (TransGen Biotech Co., Ltd., Beijing, China) according to the manufacturer’s protocol. The 2^–ΔΔCt^ method was used to measure the relative expression level of genes [65] with the endogenous control gene GhUBQ7 [66].

## 3. Results

### 3.1. Impact of Foliar Application of β-Alanine on Cotton Seedlings

**Figure 2:**
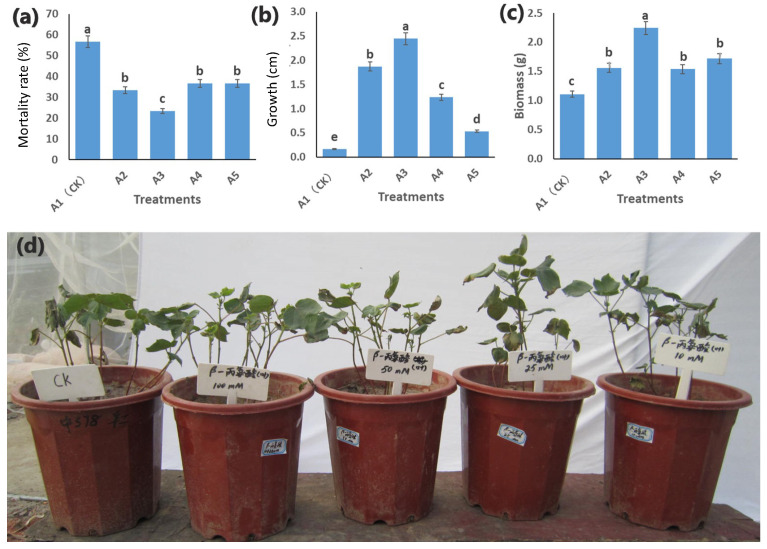
Mean comparisons of morphological characteristics, i.e., (**a**) the mortality (%), (**b**) growth (cm), and (**c**) above-ground biomass (**d**), for cotton seedlings under salt stress pre-treated with the β-alanine solution at 25 days after planting. The bar graph showed significant statistical differences (*p* = 0.05) between the treatments (A2, A3, A4, and A5) and CK (A1). Bar plots with overlapping error bars are statistically insignificant, while the letters showed statistical significance if the samples did not share letters. (**d**) Phenotypes of the cotton seedlings exposed to 0.8% salt stress after exogenous treatment (foliar application) with 100 mL of β-alanine solution in concentrations as A1(CK) = 0 mM, A2 = 10 mM, A3 = 25 mM, A4 = 50 mM, and A5 = 100 mM.

Cotton seedlings depicted different responses against the five concentrations of β-alanine. In comparison to A1(CK), the four treatment concentrations, i.e., A2, A3, A4, and A5, revealed significant differences regarding their mortality rate, i.e., A1 had the highest mortality rate, whereas the mortality rate decreased subsequently with increased β-alanine concentrations. The seedlings from A1(CK) depicted a significant decline in growth, as shown by the mean plant height (cm). However, the β-alanine-treated seedlings showed an increased plant growth under salt stress, indicating that β-alanine has helped seedlings to sustain the stress. Finally, we observed that A1(CK) had lower above-ground biomass (seedling weight in g) compared to β-alanine-treated samples. There is a similar increasing trend of above-ground biomass weight with increasing β-alanine concentrations. It has been observed that among all treatments, the A3 (25 mM) treatment behaved differently for all the traits under study, as the mortality rate for A3 was the lowest compared to all treatments. Similarly, A3 has shown a higher mean plant height and above-ground biomass than the rest of the treatments (Figure 2). These observations indicate that A3 concentration is ideal for treating cotton seedlings with β-alanine to sustain the salt stress. Nonetheless, in this study, we did not evaluate the effect of the different concentrations of β-alanine on the growth of cotton seedlings under normal (without salt) conditions. Such data could have better informed us on why A3 treatment outperformed other treatments.

### 3.2. Transcriptome Profile

In response to different concentrations of β-alanine foliar application, the transcriptome changes in cotton seedlings samples were investigated through RNA-sequencing. Fifteen independent libraries were sequenced through Illumina Hiseq, which resulted in total reads of 7.61 Gb with an average of 50,755,104 reads per library. The obtained results included average clean reads of 98.61%, GC contents of 44.71%, Q20 of 96.93%, and Q30 of 91.89% (Table S2). After filtering, we obtained approximately 818,499,314 clean reads aligned against the reference genome [52]. A total of 836,627,970 (93.99%) mapped reads were generated comprising 701,394,151 (85.78%) unique alignments and 67,649,420 (8.18%) secondary alignments from the genes of treated seedlings tissues (Table S3).

**Figure 3:**
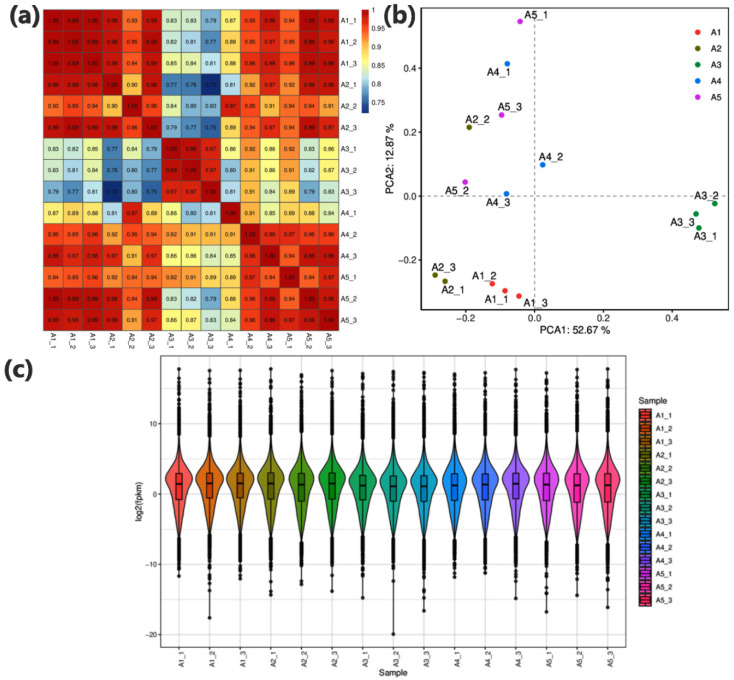
(**a**) Heatmap was for depicting Pearson’s correlation coefficient of the sample gene expression. (**b**) Principal component analysis of the sample gene expression. Different colors represent different samples. (**c**) The overall fragment/kb of transcript/million mapped reads (FPKM) values for each replicate were represented as a graph of violin boxplots. The abscissa represented different samples; the ordinate illustrated each sample expression’s log_2_ values for FPKM, where β-alanine solution in different concentrations were used as A1(CK) = 0 mM, A2 = 10 mM, A3 = 25 mM, A4 = 50 mM, and A5 = 100 mM; 1, 2, and 3 with the treatments represent the replicates.

From the 15 independent libraries, 388 new genes (Table S4) out of 67,741 total genes were identified and annotated through the following databases viz; KEGG, GO, KOG, Swiss-Prot, Pfam, and eggNOG/COG (Table S5). A trend of maximum gene expression was depicted by A1(CK) as compared to other treatments (Figure 3a). The replicates in each treatment are grouped by the Principal Component Analysis (PCA) (Figure 3b). Likewise, a range of 0.75 to 1 was observed from the Pearson correlation coefficient (PCC) analysis (Figure 3c). Hence, PCA and PCC altogether indicated that the sampling was quite reliable. The PCA results from the transcriptome profiling depicted interesting findings regarding the clear distinction of treatment A3 from the rest of the treatments by placing it on the positive side. These findings are similar to the morphophysiological results where A3 represented the better performance of cotton seedlings regarding their growth and development.

### 3.3. Identification of Differentially Expressed Genes

**Figure 4:**
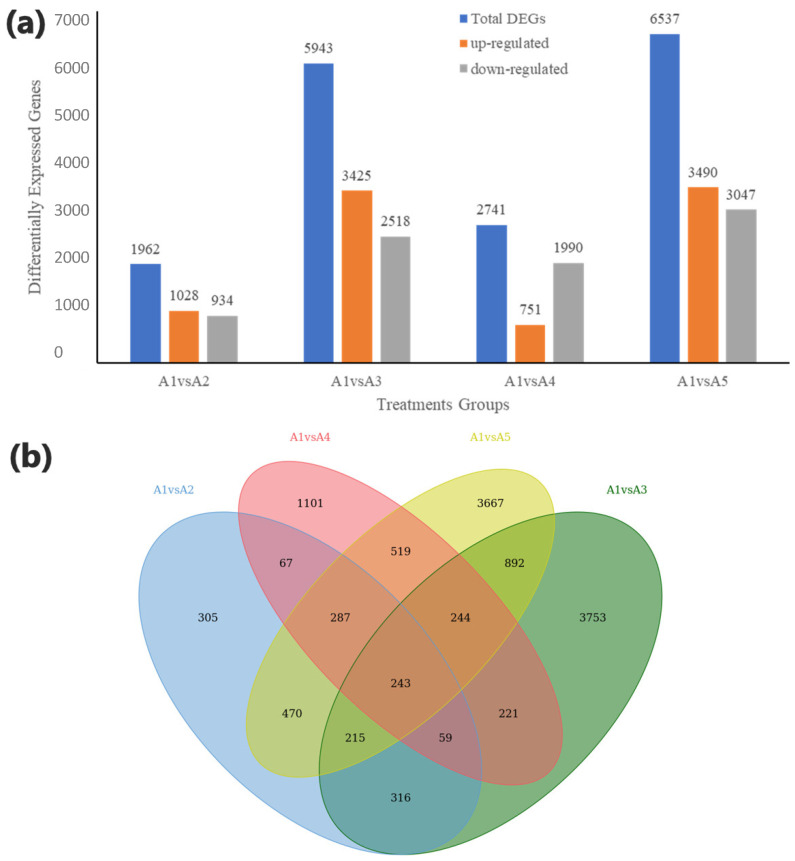
(**a**) A barplot exhibiting the number of differentially expressed genes (DEGs) with up and down regulations identified for the β-alanine-treated cotton seedling samples against salt stress. (**b**) Venn diagram illustrating the number of common and conserved DEGs between different comparison groups of treatments with CK(A1). β-alanine solution in different concentrations used were as follows: A1(CK) = 0 mM, A2 = 10 mM, A3 = 25 mM, A4 = 50 mM, and A5 = 100 mM; 1, 2, and 3 with the treatments represent the replicates.

Following are the number of differentially expressed genes (DEGs) identified in the different studied treatments as compared to CK (A1): A1 vs. A2 (1962), A1 vs. A3 (5943), A1 vs. A4 (2741), and A1 vs. A5 (6537) (Figure 4a, Table S6). Approximately 243 DEGs were observed as core conserved among all the treatment comparison groups with CK(A1), which could potentially be involved in boosting the tolerance against salinity after being affected with β-alanine treatment (Figure 4b). Furthermore, a maximum number of genes, viz., 3753 (up-regulated: 168; down-regulated: 75), were individually associated with the comparison group A1(CK) vs. A3 revealing the significant role of foliar application of 25 mM (A3) concentration of the β-alanine solution in up-regulation of tolerance in cotton. A rich amount of DEGs was discovered to be involved in plant hormone signal transduction, phenylpropanoid biosynthesis, protein processing in the endoplasmic reticulum, photosynthesis, circadian rhythm–plant, flavonoid biosynthesis, phenylalanine biosynthesis, MAPK signaling pathway, photosynthesis-antenna proteins, linoleic acid metabolism, alanine aspartate and glutamate metabolism, and β-alanine metabolism-related pathways (Table S6).

### 3.4. Functional Annotation of DEGs

The functional annotations for the identified DEGs from the comparison of CK with other treatment groups (A2, A3, A4, and A5) illustrated that about 695 and 1483 DEGs were involved in growth and development processes, respectively, 607 DEGs were involved in salt stress tolerance, 283 DEGs in photosynthesis, 212 and 138 in the regulation of enzymes, i.e., abscisic acid (ABA) and auxin synthesis, respectively.

#### 3.4.1. Germination, Growth and Development Related DEGs

About 251 DEGs were related to the production of SAUR proteins (ko: K14488) involved in regulating germination, growth, development, and auxin (IAA) production. These genes (*gene-LOC107886087*, *gene-LOC107890656*, *gene-LOC107893912*, *gene-LOC107896513*, *gene-LOC107909660*, *gene-LOC107919496*, *gene-LOC107935792*, *gene-LOC107948898*, *gene-LOC121212455*, *gene-LOC121225065*) were up-regulated in comparison group A1 vs. A3 (25 mM) of β-alanine treatment. Several DEGs (41) encode RAB11A proteins (ko: K07904) related to growth, development, and ABA production. The *gene-LOC107930441* encoding this protein was found with exclusive expression in the treatment comparison group A1 vs. A5. The PYL protein (ko: K14496) was encoded by a total of 40 DEGs that regulate ABA enzyme production. Following DEGs, viz., *gene-LOC107929605*, *gene-LOC107930169*, *gene-LOC107931756*, *gene-LOC107937632*, *gene-LOC107950128*, *gene-LOC121204798*, and *gene-LOC121222372* were discovered as down-regulated except *gene-LOC107930169,* which was up-regulated only in the treatment comparison group A1 vs. A5. These DEGs are associated with ABA production, and ABA is a growth-inhibiting enzyme. In salt stress conditions, tolerant seedlings need to grow without adversely affecting their growth and development. Similarly, CPK protein (ko: K13412) and AHK2_3_4 protein (ko: K14489) were encoded by 22 and 24 DEGs, respectively, involved in the regulation of the growth and development of stressed seedlings. The CPK protein was encoded by *gene-LOC107912990* and *gene-LOC107909452*, which were down-regulated in the stress condition to promote growth and development. The AHK2_3_4 protein was encoded by *gene-LOC107924440* and *gene-LOC107913518* DEGs that displayed up-regulation expression patterns to enhance germination, growth, and development. The PIN proteins (ko: K13947) were encoded by 32 DEGs participating in regulating the developmental process and production of IAA. Following DEGs *gene-LOC121211249*, *gene-LOC107934295*, *gene-LOC107931052*, *gene-LOC107922128*, *gene-LOC107910425*, and *gene-LOC107904680* expressed themselves with up-regulation to enhance development and IAA production in stress condition after foliar application of the β-alanine solution. The SIAH1 proteins (ko: K04506) encoded by 26 DEGs were involved in developing seedlings under stress conditions. The DEGs *gene-LOC107940377*, *gene-LOC107937458*, and *gene-LOC107935481* revealed up-regulation in the expression patterns for enhancement of development after β-alanine treatment. The RAPTOR (ko: K07204) protein involved in the growth and development was encoded by 25 DEGs. Following DEGs *gene-LOC121226555*, *gene-LOC121203258*, *gene-LOC107953298*, *gene-LOC107944816*, *gene-LOC107939829*, *gene-LOC107936182*, *gene-LOC107935978*, *gene-LOC107923849*, *gene-LOC107914561*, *gene-LOC107903529*, *gene-LOC107896885*, and *gene-LOC107894029* revealed their exclusive expression patterns to enhance growth and development in salt stress challenged growing environment (Table S7).

#### 3.4.2. Salt Stress-Related DEGs

The HSP20 proteins (ko: K13993) were encoded by 40 DEGs with a general trend of down-regulation in their expression, particularly in the A1 vs. A3 comparison group that might be involved in tolerance against salt stress. In the rest of the treatment comparison groups, the protein-coding DEGs *gene-LOC107886383* and *gene-LOC107890212* showed up-regulation to enhance salt tolerance. The MSI proteins (ko: K14411) were encoded by 27 DEGs and showed their involvement in salt stress tolerance. These DEGs are listed as *gene-LOC107944246*, *gene-LOC107905691*, *gene-LOC107962067*, *gene-LOC107956237*, *gene-LOC107934010*, *gene-LOC107917827*, *gene-LOC121215240*, and *gene-LOC107932143*, and showed involvement in the salt stress for cotton seedlings. CPK protein (ko: K13412) was discovered with 14 DEGs under stress conditions related to salt stress. The DEG *gene-LOC107891295* gave an up-regulated expression pattern to enhance tolerance against salt stress. AHK2_3_4 protein (ko: K14489) was encoded by 10 DEGs related to salt stress. The DEG *gene-LOC107924440* showed up-regulation to boost salt stress tolerance in the cotton seedlings in the stress condition after foliar application with the β-alanine solution.

#### 3.4.3. Photosynthesis Related DEGs

**Figure 5:**
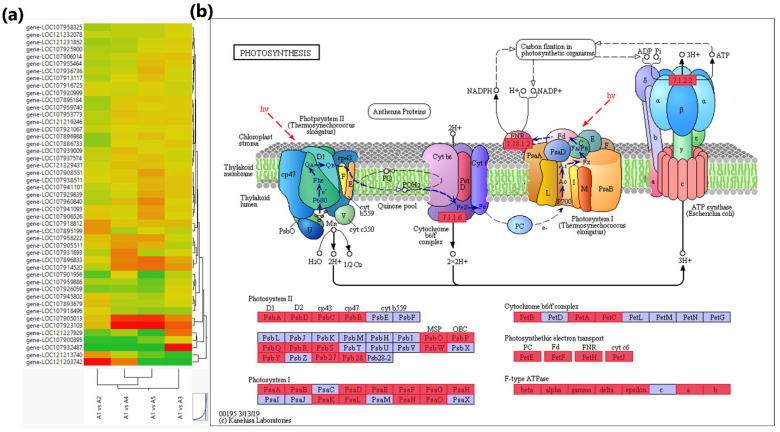
(**a**) Two-way hierarchical clustering of DEGs enriched in photosynthesis pathways (Ko00195) based on log_2_FC values. KEGG pathway map of the DEGs enriched in photosynthesis-antenna proteins (Ko00195). The red boxes are for the representation of enriched DEGs in photosynthesis. (**b**) The pathway showed increased expression of DEGs for photosystem II, chlorophyll, antenna proteins, etc., in the β-alanine-treated seedlings from A1 vs. A3 treatment as compared to A1(CK). β-alanine solution in different concentrations used were as follows: A1(CK) = 0 mM, A2 = 10 mM, A3 = 25 mM, A4 = 50 mM, and A5 = 100 mM; 1, 2, and 3 with the treatments represent the replicates.

Approximately 73 different types of proteins encoded by 283 DEGs were discovered to regulate the photosynthesis process in response to the β-alanine treatment of cotton seedlings. The encoded proteins mainly include HAO (ko: K11517), psbQ (ko: K08901), E2.4.1.14 (ko: K00696), ppc (ko: K01595), petH (ko: K02641), psbP (ko: K02717), HMOX, hmuO, ho (ko: K00510), LHCB (ko: K08912), HPR2 (ko: K15919), psaD (ko: K02692), FTH (ko: K00522), SLC35E1 (ko: 15283), ATPF1D (ko: K02113), and many others. Out of these, 47 DEGs were enriched in the photosynthesis pathway. Six photosystem II oxygen-evolving enhancer protein 3 (PsbQ) encoded by *gene-LOC107918812* and *gene-LOC107895199*, five photosystem II oxygen-evolving enhancer protein 2 (PsbP) encoded by *gene-LOC107896833* and *gene-LOC107914520*, four photosystem II 22 kDa protein (PsbS) controlled by *gene-LOC107905013* and *gene-LOC107923103*, three ferredoxin–NADP^+^ reductases (PetH), two F-type H^+^-transporting ATPase (ATPF0B, atpF), two plastocyanins (PetE), two ferredoxin (PetF), two photosystem I subunit IV (PsaE), two photosystem I subunit III (PsaF), two photosystem I subunit VI (PsaH), two photosystem I subunit X (PsaK), two photosystem II oxygen-evolving enhancer protein 1 (PsbO), two photosystem II 10 kDa protein (PsbR) regulated by *gene-LOC121213740* and *gene-LOC121203742*, two photosystem II 6.1 kDa protein (PsbW), two photosystem II PsbY protein (PsbY), a F-type H^+^-transporting ATPase subunit γ (ATPF1G, atpG), a cytochrome b6-f complex iron-sulfur subunit (PetC) (*gene-LOC107886733*), a cytochrome c6 (PetJ) (*gene-LOC107900395*), a photosystem I subunit XI (PsaL) encoded by *gene-LOC107895184*, a photosystem I subunit (PsaO) controlled by *gene-LOC107960840*, a photosystem II Psb27 protein (Psb27), and a photosystem II D1 protein (PsbA) (*gene-LOC121227929*) were mostly up-regulated in β-alanine-treated seedlings as compared to A1(CK) (Figure 5, Table S7).

### 3.5. Transcription Factors

Approximatively 1281 transcription factors (TFs) responded to β-alanine treatment of cotton seedlings and were related to growth, development, photosynthesis, salt stress tolerance, and ABA and IAA biosynthesis regulation. The TFs related to the mentioned functions included *MYBP*, *HD*-*ZIP*, *RPB*, *ARF*, *PCBP*, *MYC*, *ELF*, *EREB*, *DELLA*, *SMARCC*, *NFYA*, *MEF*, *ABF*, *H2A*, *H4*, *WRKY*, and *HK* (Table S8). These results indicate that TFs play a major role in the regulation and enhancement of salinity tolerance in the β-alanine-treated seedlings compared to A1(CK).

### 3.6. qRT-PCR Validation of Selected DEGs

We performed a qRT-PCR experiment by selecting 10 DEGs and evaluated their transcripts levels under A1 vs. A3 treatments. As shown in Figure S1, all five genes displayed differential expression between A1 and A3. A total of five genes were up-regulated while the remaining were down-regulated in A1 vs. A3, exactly as observed in the RNA-seq data. We further performed a Pearson correlation analysis between the two datasets (qRT-PCR and RNA-seq), which resulted in a high correlation score (R^2^ = 0.84). This result indicates that the gene expression profile from the RNA-seq analysis is reliable.

### 3.7. Metabolome Profiling

**Figure 6:**
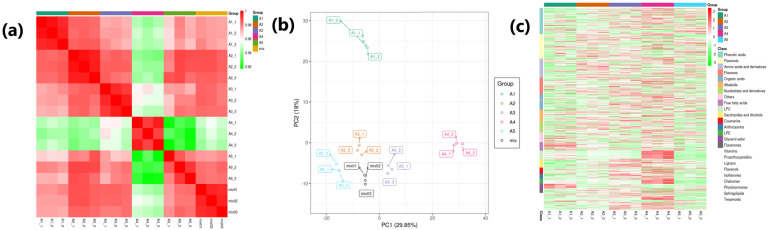
(**a**) Pearson’s correlation coefficient heatmap of the detected metabolites in β-alanine-treated and control cotton seedlings challenged with salt stress. (**b**) Principal component analyses of metabolites extracted from CK and treated cotton seedlings. (**c**) Heatmap of relative intensities of metabolites. Where A1(CK) = 0.8% salt stressed, A2, A3, A4, and A5, salt-stressed seedlings exogenously sprayed with β-alanine solution in different concentrations of A2 = 10 mM, A3 = 25 mM, A4 = 50 mM, and A5 = 100 mM; 1, 2, and 3 with the treatments represent the replicates.

**Figure 7:**
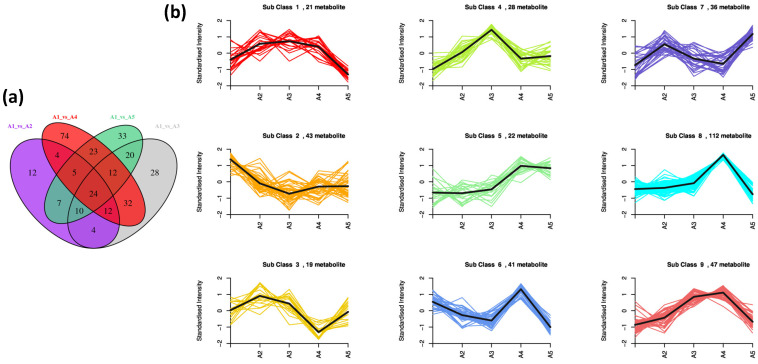
(**a**) Venn diagram of the differentially accumulated metabolites between different treatment comparisons. (**b**) K-means diagram of the differentially accumulated metabolites among CK and treated cotton seedling samples (A1 vs. A2, A1 vs. A3, A1 vs. A4, and A1 vs. A5). The *x*-axis represents the sample groups, the *y*-axis represents the relative content of standardized metabolites, the sub-class represents the number of the metabolite category with the same changing trend, and the metabolite represents the number of metabolites in the category. Where A1(CK) = 0.8% salt stressed, A2, A3, A4, and A5, salt-stressed seedlings exogenously sprayed with β-alanine solution in different concentrations of A2 = 10 mM, A3 = 25 mM, A4 = 50 mM, and A5 = 100 mM; 1, 2, and 3 with the treatments represent the replicates.

Almost 735 metabolites were identified in response to the β-alanine treatment of cotton seedlings (Table S9). The Pearson’s correlation coefficient (PCC) observed for the treated cotton seedling samples was in a range of 0.92 to 1.00, endorsing the reproducibility of biological replicates from CK and treatments (Figure 6a). The PCA illustrated replicates of each treatment as an individual group, separating from other treatments. The two PCs covered almost 47.85% of the total variation (Figure 6b). These metabolites from all the seedling samples (CK and treated) could be classified into 25 classes. The phenolic acids compounds were found in higher amounts in all the studied seedlings, followed by flavonols, amino acids and derivatives, and flavones (Figure 6c). A total of 540 metabolites were observed with differential accumulation between CK and β-alanine treatment groups. We identified 78, 142, 186, and 134 differentially accumulated metabolites (DAMs) in A1 vs. A2, A1 vs. A3, A1 vs. A4, and A1 vs. A5, respectively (Table S10). Among these metabolites, 24 DAMs were commonly shared by all four mentioned groups of treatment samples (Figure 7a). The clustering through K-Means analysis unraveled nine clusters of metabolites with different accumulation trends in the samples (Figure 7b).

The differentially accumulated metabolites were enriched in ABC transporters, pentose and glucuronate interconversions, lysine degradation, galactose metabolism, tryptophan metabolism, arginine biosynthesis, metabolic pathways, biosynthesis of secondary metabolites, biosynthesis of amino acids, glutathione metabolism, phenylpropanoid biosynthesis pathways, and tropane, piperidine and pyridine alkaloid biosynthesis pathways in the β-alanine-treated cotton seedling samples in comparison to A1(CK) (Table S10). The common DAMs observed were mostly alkaloids, amino acids, and derivatives (1,2-N-Methylpipecolic acid, Cyclo (Pro-Pro), Proline betaine, coumarins, organic acids, saccharides, and alcohols, which were highly accumulated in the treated seedlings as compared to A1(CK) revealing their significant roles in enhancing tolerance against stress. However, the free fatty acids, flavones, flavonols, glycerol esters, lignans, and some phenolic acids, were observed as less accumulated in β-alanine-treated cotton seedling samples compared to A1(CK) (Table 1).

### 3.8. Treatment Specific Metabolites

**Figure 8:**
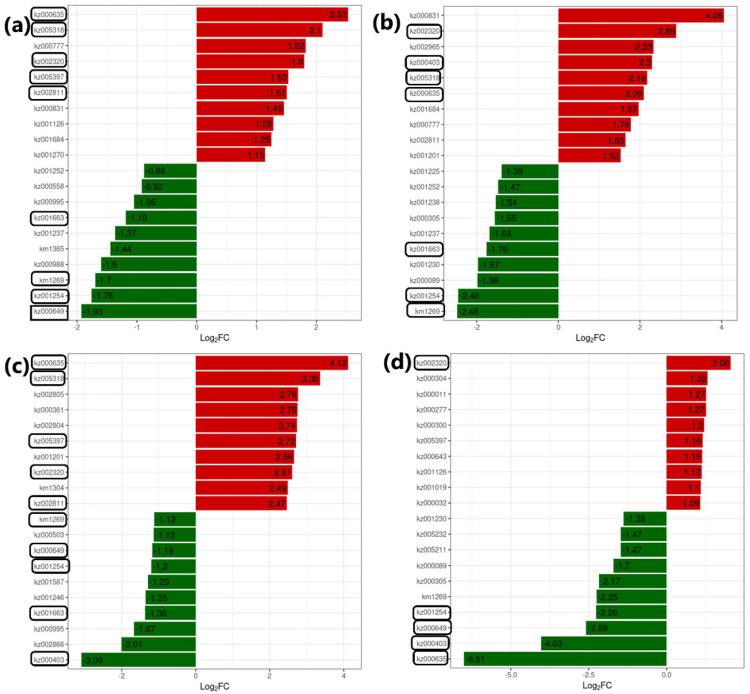
Top 10 up-down accumulated metabolites in different treatment comparisons of the β-alanine-treated cotton seedlings exposed to salt stress. (**a**) A1vsA2, (**b**) A1vsA3, (**c**) A1vsA4, (**d**) A1vsA5. The bar graphs with log_2_ fold change values of the differentially accumulated metabolites, where red and green colors represent up and down regulations, respectively and the encircled metabolite compounds were found conserved in more than two comparison groups of treatments. A1(CK) = 0.8% salt stressed, A2, A3, A4, and A5 = 0.8% salt-stressed seedlings with foliar application of 100 mL of 50 µM, 100 µM, 200 µM, and 500 µM β-alanine solution, respectively.

Out of 78 A2-specific DAMs, 47 metabolites were highly accumulated in the β-alanine-treated samples of group A2 compared to A1(CK). Most of the highly accumulated metabolites from A2 were amino acids and derivatives, followed by alkaloids (Table S10). The top 10 metabolites with differential accumulation in A2 compared to A1(CK) are illustrated in Figure 8a. From the 142 A3-specific metabolites, 93 DAMs were highly up-regulated in A3-treated seedling samples compared to A1(CK). They were mainly from saccharides, alcohols, organic acids, coumarins, and amino acids and derivatives (Figure 8b). A total of 151 DAMs out of 186 metabolites were highly up-regulated in A4-treated seedling samples compared to A1(CK). Most of the top accumulated metabolites were from phenolic, organic, LPC, and LPE compounds (Figure 8). From the 134 A5-specific DAMs, 44 metabolites were up-regulated compared to A1(CK). The top differentially accumulated ones include phenolic, organic, amino, and derivatives. Among the top highly accumulated DAMs, kz002320 (N,N’-Diferuloylputrescine) and kz005318 (7-Methoxy-5-Prenyloxycoumarin) were discovered in almost all the treatment groups seedlings and could be characterized by increased antioxidative activity in cotton seedlings (Table S10). This compound has been reported earlier for its antioxidative activity [67,68]. However, among the top less accumulated metabolites, kz001254 (9,10,13-Trihyroxy-11-octadecadienoic acid) and kz000649 (1,7-Dimethylxanthine) were found in all comparison groups’ seedlings.

### 3.9. Conjoint Analysis

**Figure 9:**
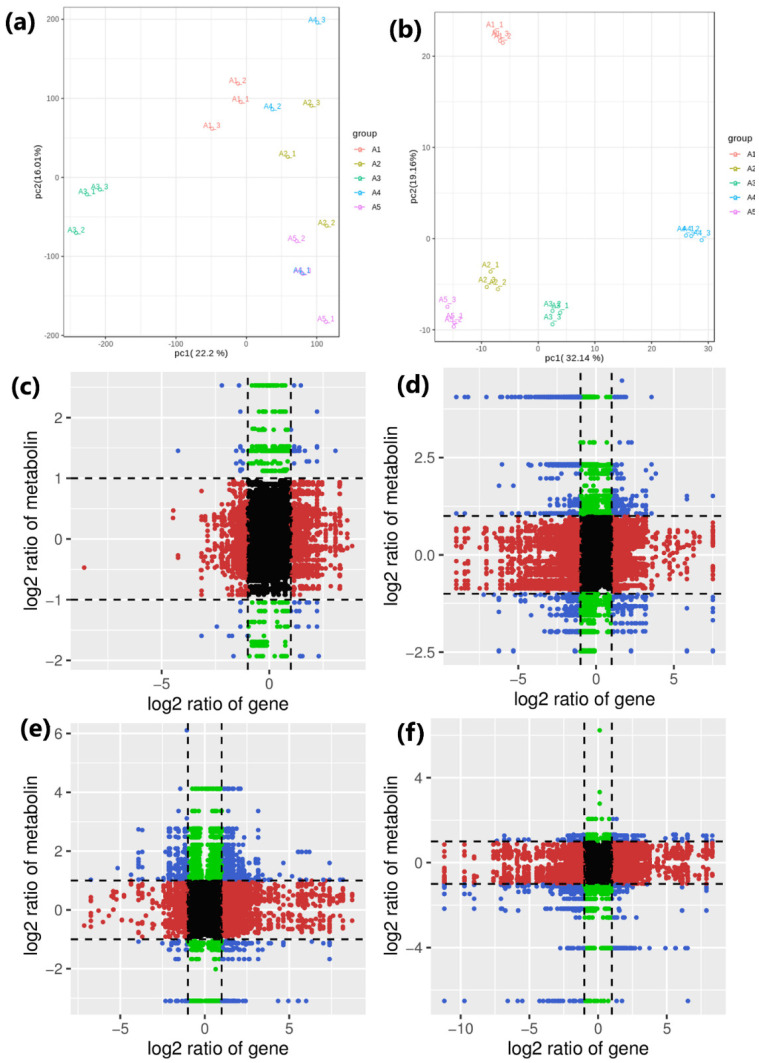
Conjoint analyses of transcriptome and metabolome data from β-alanine-treated cotton seedling samples. PCA of (**a**) transcriptome and (**b**) metabolome data to visualize the differences between the sample groups and no differences between replicates. Pearson correlation coefficient analyses via nine quadrant plots of genes and metabolites illustrate different multiples of genes and metabolites with a correlation coefficient >0.8 in each differential group. (**c**) A1vsA2, (**d**) A1vsA3, (**e**) A1vsA4, (**f**) A1vsA5. The black dotted line, from left to right, from top to bottom, divides the chart into 9 quadrants. A1(CK) = 0.8% salt stressed, A2, A3, A4, and A5 = 0.8% salt-stressed seedlings with foliar application of 100 mL of 50 µM, 100 µM, 200 µM, and 500 µM β-alanine solution, respectively.

Both transcriptome and metabolome data were integrated and statistically analyzed to examine the relationship between genes and metabolites at different levels. Based on PCA scatterplots, the triplicated sample groups were separated, and the samples from treatment showed a distinct place from other non-treated (CK-A1) samples in metabolites and transcriptome data results (Figure 9a,b). The differential genes and differential metabolites of the same group were simultaneously mapped to the KEGG pathway diagram to understand the relationship between genes and metabolites. A total of 3363 DEGs were discovered in association (Pearson’s correlation coefficient > 0.8) with 623 metabolites, with most of them jointly controlling the regulation of single or multiple metabolites (Figure 9). The highest number of DEGs (1519) was associated with the compound glutathione reductase-GR (kz000403); similarly, a significant amount of DEGs (580) were associated with the compound L-Cysteine-Cys (kz000005). GR and Cys are considered among the important antioxidant enzymes in plants. GR enzyme catalyzes the reduction of glutathione disulfide to reduced glutathione with the accompanying oxidation of NADPH and has been reported to have a key role in salt stress [69,70]. This DAM depicted up-regulation in A1 vs. A3 while showing down-regulation in A1 vs. A4 and A1 vs. A5. Cys dropped down the peroxidase activity, proline, and total sugars, indicating that it has reductant characteristics. Cys declined the oxidative stress in salt-stressed growing medium with its significantly higher accumulation in A1 vs. A5 β-alanine treatment samples representing salt tolerance (Table S11). Thus, the capacity of GR and Cys to hold redox reactions during stress is crucial against adverse effects created by salt toxicity to enhance growth and development of seedlings. These results have been validated by the correlation network diagrams revealing their relationships with DEGs encoding the proteins involved in salt tolerance pathways (Figure S2).

## 4. Discussion

β-alanine pretreatment enhances tolerance against salinity in cotton seedlings.

β-alanine is among those 250 amino acids that plants usually synthesize for several purposes like anti-microbial, toxins against invertebrates and vertebrates, anti-herbivory, in response to abiotic stresses, and nitrogen storage, etc. [71,72]. Generally, this non-proteinogenic amino acid is known for human studies. It participates in the synthesis of vitamin B5 (pantothenate) [73] in almost all organisms and plants as well, which is utilized in the formation of “Coenzyme A” and acyl-carrier protein [32,74]. Among the several unique functions associated with this β-alanine in plants, the prominent one is its accumulation as a standard response molecule to abiotic stress including salinity, drought, elevated temperature, and heavy metal shock [32,75]. The current study explored the significant roles of β-alanine in cotton plants’ response to salinity stress at the seedling stage. The results revealed that β-alanine pretreatment enhances tolerance against salinity in cotton seedlings, consistent with previous findings [3,76].

Besides the universal significance of this non-proteinogenic β-alanine acid in plants for the biosynthesis of CoA, phospholipids, and fatty acids, it is also involved in the secondary metabolism operations such as biosynthesis of lignin as well as in producing various types of stress-responsive molecules. The metabolomic profiling of *A. thaliana* indicated higher accumulations of β-alanine and other compounds like polyamine putrescine and L-alanine in response to heavy metal stress (cadmium ion) [77]. In *Medicago truncatula*, *Vigna unguiculata*, and *A. thaliana*, the β-alanine level was significantly elevated against several stresses, particularly heat shock and salinity [71,72,74,78]. In wheat species and sea anemone species, during salt stress, the accumulation of β-alanine was increased along with its synthesis from aspartic acid and a decrease in its oxidation rate [2,32,76].

β-alanine pretreatment altered the transcriptome in cotton seedlings.

Tolerance to salinity is a phenomenon of controlled expression displayed by several coordinated genes and the subsequent regulatory metabolic and signaling pathways. A considerable number of DEGs were observed in current findings of β-alanine-treated seedling samples related to the regulation of morpho-physiological pathways for enhancing salt stress tolerance. The small auxin up RNA “*saur*” gene (ko: K14488) encodes auxin (IAA) protein [79]. In the current study, up-regulation of this gene is observed through the involvement of IAA during the production and enhancement of growth and development process. Such a role of this protein family was consistent with previous studies on rice [80], *Arabidopsis* [81,82], and other plants [79] for auxin production, growth promotion, and enhanced development under various environmental stress conditions [83,84]. Heat shock protein gene “*hsp20*” was also discovered among DEGs for the enhancement of salinity tolerance along with enhanced growth and development in the β-alanine-treated cotton seedling samples. Similar function of this gene was discovered in rice [85], African bermudagrass [86], potato [87], and grape [88] for salinity and heat tolerance. Another gene discovered among DEGs was pyrabactin resistance 1-like (*pyl*), which encodes the core protein involved in regulating the signaling network of ABA in response to some abiotic stresses. Abscisic acid biosynthesis decreased in the salt-stressed environment of *Cucumis sativus*, implying that the ABA receptor stopped responding or gave reduced expression in a stressed environment. Alike findings related to the reduced expression of the *pyl* gene were also observed in the current study depicted by the β-alanine-treated cotton seedlings challenged with NaCl stress [89]. Similar findings were described earlier related to the functions of this gene in *Brassica napus* [90], maize, and cotton [91] for mediating abiotic stress responses. Salt and drought resistance mechanisms were also earlier reported in association with creatine phosphokinase *cpk* gene in *A. thaliana* [92] and *B. napus* [93] that are in line with our results. Another discovered protein in our findings is *Arabidopsis* histidine kinase protein encoded by *AHK2_3_4* with biological process GO annotation of cytokinin-activated signaling pathway. It has earlier been reported in plants for regulation of salinity tolerance mechanisms [94] and in *Arabidopsis* for freezing tolerance [95,96]. Another protein named “auxin efflux carrier family protein” encoded by *pin* gene (ko: K13947) was previously reported for controlling growth and development under environmental stress in *Arabidopsis* [97]. A DEG for “E3 ubiquitin-protein ligase *siah1* protein” has been reported with involvement in the signal transduction pathway and MAPK signaling pathway [98]. Some DEGs discovered for encoding regulatory associated protein of mTOR “RAPTOR” for enhanced growth and development in the β-alanine-treated seedlings. These proteins were previously reported for environmental adaptation in multiple species and membrane trafficking [99,100,101].

The DEGs that enhanced the photosynthesis process in treated seedlings were also identified. The hydroxy-acid oxidase encoding gene “*hao*” discovered in our study was earlier reported as involved in the biosynthesis of secondary metabolites, metabolic pathways, glyoxylate and dicarboxylate metabolism, and oxidoreductases [102]. Similarly, light-harvesting chlorophyll a/b “*lhc*” genes and photosystem-II subunit “*psb*” genes were reported previously in reed ecotypes [103], in xanthophylls [104], and in *Arabidopsis* [105] for encoding abiotic stress proteins, particularly salinity and drought. Furthermore, the up-regulation in the production of proteins, i.e., HMOX, hmuO, ho, and psaD, illustrated that biosynthesis of chlorophyll increased in the β-alanine-treated seedlings validated by the expression of antenna proteins (LHCB) that helped to mitigate the adverse effects of the saline environment. The relationship of antenna proteins with salt tolerance was earlier supported by a study in *Arabidopsis* [106]. Transcription factors (TFs) play pivotal roles in every regulatory and metabolic pathway related to tolerance of salt stress and other abiotic stresses. Several studies have been reported on the diverse roles of TF families like *WRKY*, *HD-ZIP*, *MYB*, *MYC*, and zinc-finger genes controlling several regulatory pathways involved in stress tolerance [107,108]. In a study on maize, the seed germination in a salt-stressed medium was rescued with overexpression of positive responsive salinity genes “*EREB*” by regulating ABA, GA, and ROS scavenging pathways with pretreatment [109]. Over-expression of *wrky* genes in wheat and *myb* genes in Medicago [110] were observed under salt stress conditions by acting as a positive regulator of salt stress via increasing osmotic adjustments and enhancing membrane stability [111]. It elucidates that the seedlings in the current study also showed the overexpression of *WRKY*, *EREB*, and other expressed TFs genes to cope with stress conditions.

Various metabolic pathways were impacted by β-alanine pretreatment in cotton seedlings.

On treatment with β-alanine, the seedling extract revealed increased amounts of different metabolites such as alkaloids, coumarins, organic acids, amino acids and derivatives, saccharides, and alcohols. In previous reports, it has been extensively elaborated that amino acids (proline, alanine, proline-betaine), polysaccharides, and organic acids, accumulate in higher amounts to play significant roles in osmotic adjustments [112], protecting the enzymes [113] and intracellular structures and mitigating the oxidative stress damages in salt stress response [114,115]. Accumulation of two or more amino acid groups leads to the formation of polyamines due to polyvalent binding, which is usually stimulated by osmotic stress, nutrient deficiency, low pH, potassium deficiency, and/or less light availability to plants [115,116]. The significant roles of polyamines include development, growth, regulation of signals, gene expression, and tolerance against stresses [117] by reactive oxygen species (ROS) homeostasis [118]. Studies on *M*. *crystallinum*, *A. thaliana*, and *Thellungiella salsuginea* revealed greater accumulations of polyamines in the halophytes in salt stress. This and many other studies illustrated that the polyamines, organic acids, and phenolic compounds scavenge free radicals, followed by activation of antioxidants to keep ROS at lower amounts in plant tissues [117,119,120].

Conjoint analysis of metabolome and transcriptome data revealed glutathione reductase-GR (kz000403) and L-Cysteine-Cys (kz000005) as prominent metabolites associated with the highest number of DEGs, i.e., 1519. In earlier reports, this metabolite is speculated to have a significant association with tolerance against salinity. In rice, the gene encoding this metabolite “GR” was knocked out to produce mutant rice for validation of GR’s role in salt tolerance [121]. This metabolite protein is localized in the mitochondria and chloroplast of rice and when the mutant rice was grown under salt stress, the GR metabolite gave no expression in the mitochondria and chloroplast; thus, the overall tolerance of that mutant rice plant got reduced by 20%. In other studies, the role of GR has been proved to be positively associated with abiotic stresses, particularly salinity [69,122]. Cys is an amino acid and precursor in the formation of various biomolecules (Fe-S clusters, vitamins, or cofactors) [123,124] and biocomponents (polyamines, glutathione, methionine, and glucosinolates) [125]. GR and Cys are considered as significant elements of antioxidant machinery that plants utilize against abiotic stresses as this enzyme catalyzes the reduction reaction of glutathione disulfide to glutathione and NADPH oxidation [69,125]. These reactions are eminent in maintaining redox balance within plant cells [69,70,126]. Thus, it implies that treatment with β-alanine enhanced the positive regulation of GR and Cys, which helped to maintain ROS balance inside the cell by mitigating oxidative stress damage.

## 5. Conclusions

Foliar spraying with 25 mM β-alanine solution (A3) improved cotton seedlings tolerance against salinity stress. Transcriptome analysis revealed several genes in association with positive regulation of abiotic/salt tolerance, growth, and development processes and their related signaling pathways in the β-alanine-treated seedlings as compared to untreated plants. Highly expressed transcription factors observed were *MYBP*, *HD*-*ZIP*, *ARF*, *MYC*, *EREB*, *DELLA*, *ABF*, *H2A*, *H4*, *WRKY*, and *HK* involved in the positive regulation and enhancement of salinity tolerance in treated seedlings. Similarly, the higher accumulation of polyamines, coumarins, fatty acids, organic acids, and phenolic compounds also facilitated the tolerance mechanism by positively regulating the metabolic pathways involved in ROS scavenging. Overall, we propose that cotton’s salt tolerance and improved productivity can be managed by foliar application of β-alanine at the seedling stage.

## Figures and Tables

**Table 1:** List of core conserved 24 DAMs related to the treatment comparison groups of CK(A1) and β-alanine-treated cotton seedlings.

Index	Class	A1 vs. A2	A1 vs. A3	A1 vs. A4	A1 vs. A5	Compound Name
kz002320	Alkaloids	up	up	up	up	N,N’-Diferuloylputrescine
kz000335	Amino acids and derivatives	up	up	up	up	1,2-N-Methylpipecolic acid
kz005397	Amino acids and derivatives	up	up	up	up	Cyclo(Pro-Pro)
kz000337	Amino acids and derivatives	up	up	up	up	Proline betaine (ProBet)
kz005318	Coumarins	up	up	up	up	7-Methoxy-5-Prenyloxycoumarin
kz001177	Coumarins	up	up	up	down	Scoparone
kz000635	Nucleotides and derivatives	up	up	up	down	Thymine
kz002811	Organic acids	up	up	up	up	2-hydroxyhexadecanoic acid
kz000297	Organic acids	up	up	up	up	Oxoadipic acid
kz000499	Phenolic acids	up	up	up	up	Trans-4-Hydroxycinnamic Acid Methyl Ester
kz001126	Saccharides and Alcohols	up	up	up	up	Ribitol
kz000649	Nucleotides and derivatives	down	down	down	down	1,7-Dimethylxanthine
kz001237	Free fatty acids	down	down	down	down	12,13-EODE
kz001254	Free fatty acids	down	down	down	down	9,10,13-Trihyroxy-11-octadecadienoic acid
kz001246	Free fatty acids	down	down	down	down	9,10-Dihydroxy-12-octadecenoic acid
kz001238	Free fatty acids	down	down	down	down	9,10-EODE
kz001252	Free fatty acids	down	down	down	down	9,12,13-Trihyroxy-10,15-octadecadienoic acid
kz001230	Free fatty acids	down	down	down	down	9-HOTrE
km1269	Flavonols	down	down	down	down	Di-O-methylquercetin
kz002805	Others	down	up	up	down	Hemigossypol
km1365	Glycerol ester	down	down	down	down	MAG (18:4) isomer1
kz000503	Phenolic acids	down	down	down	down	Methyleugenol
kz001663	Flavones	down	down	down	down	norwogonin
kz000995	Lignans	down	down	down	down	Syringaresinol

## Data Availability

The raw RNA-seq data have been submitted to NCBI SRA under the project number: PRJNA856789 (https://www.ncbi.nlm.nih.gov/bioproject/?term=PRJNA856789, accessed on 11 May 2023).

## Supplementary Materials

The following supporting information can be downloaded at: https://www.mdpi.com/article/10.3390/genes14091825/s1. Figure S1. qRT-PCR validation of selected genes between A1 and A3 treatments and correlation analysis with RNA-seq data. The genes highlighted in blue and red are those down- and up-regulated, respectively; Figure S2. Differential metabolites to differential genes network diagram; Table S1. List of primer sequences for the selected genes used for the qRT-PCR experiment; Table S2. Summary of raw and clean reads from transcriptome profiling of β-alanine-treated cotton seedling samples; Table S3. Summary of RNA-seq data obtained from transcriptome profiling of β-alanine treated cotton seedling samples; Table S4. List of expressed new genes with FPKM values for individual replicates at different time points; Table S5. List of functional annotations related to expressed genes from different databases; Table S6. List of differentially expressed genes (DEGs) and functional annotations in different comparison groups of CK and β-alanine treated cotton seedlings samples; Table S7. List of selected DEGs based on their relevant functions in the β-alanine-treated cotton seedling samples; Table S8. List of transcription factors with their relevant functions in the β-alanine-treated cotton seedling samples; Table S9. List of metabolites identified through the UPLC-MS/MS analyses in response to β-alanine treatment of cotton seedlings along with their expressions and functional annotation; Table S10. List of differentially accumulated metabolites (DAMs) related to the different comparison groups of CK and β-alanine treated cotton seedling samples; Table S11. List of associated DEGs and DAMs from the different comparison.
