# Supplementary material for: Integrated Transcriptome and Metabolome Analysis of Salinity Tolerance in Response to Foliar Application of β-Alanine in Cotton Seedlings
Source: Genes (Basel). 2023 Sep 20;14(9):1825. doi: 10.3390/genes14091825 (PMC10531431; doi:10.3390/genes14091825)
Supplement: Supplementary file 1 [file genes-14-01825-s001.zip › Figure S1.pptx]

## Slide 1
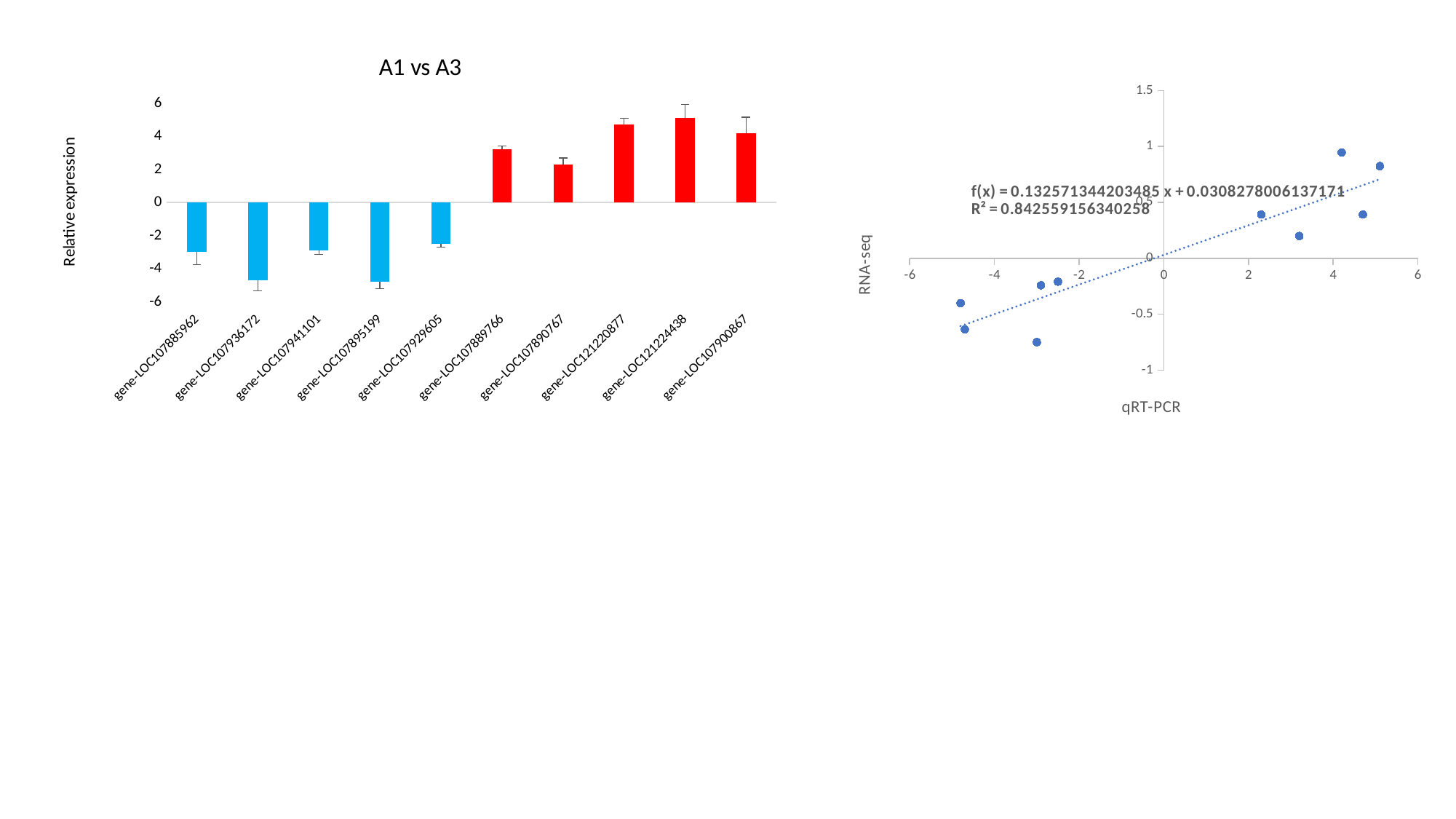

### Chart: A1 vs A3
| Category | |
|---|---|
| gene-LOC107885962 | -3.0 |
| gene-LOC107936172 | -4.7 |
| gene-LOC107941101 | -2.9 |
| gene-LOC107895199 | -4.8 |
| gene-LOC107929605 | -2.5 |
| gene-LOC107889766 | 3.2 |
| gene-LOC107890767 | 2.3 |
| gene-LOC121220877 | 4.7 |
| gene-LOC121224438 | 5.1 |
| gene-LOC107900867 | 4.2 |
### Chart
| Category | |
|---|---|
