# Supplementary figures and images for: Integrated Transcriptome and Metabolome Analysis of Salinity Tolerance in Response to Foliar Application of β-Alanine in Cotton Seedlings
Source: Genes (Basel). 2023 Sep 20;14(9):1825. doi: 10.3390/genes14091825 (PMC10531431; doi:10.3390/genes14091825)

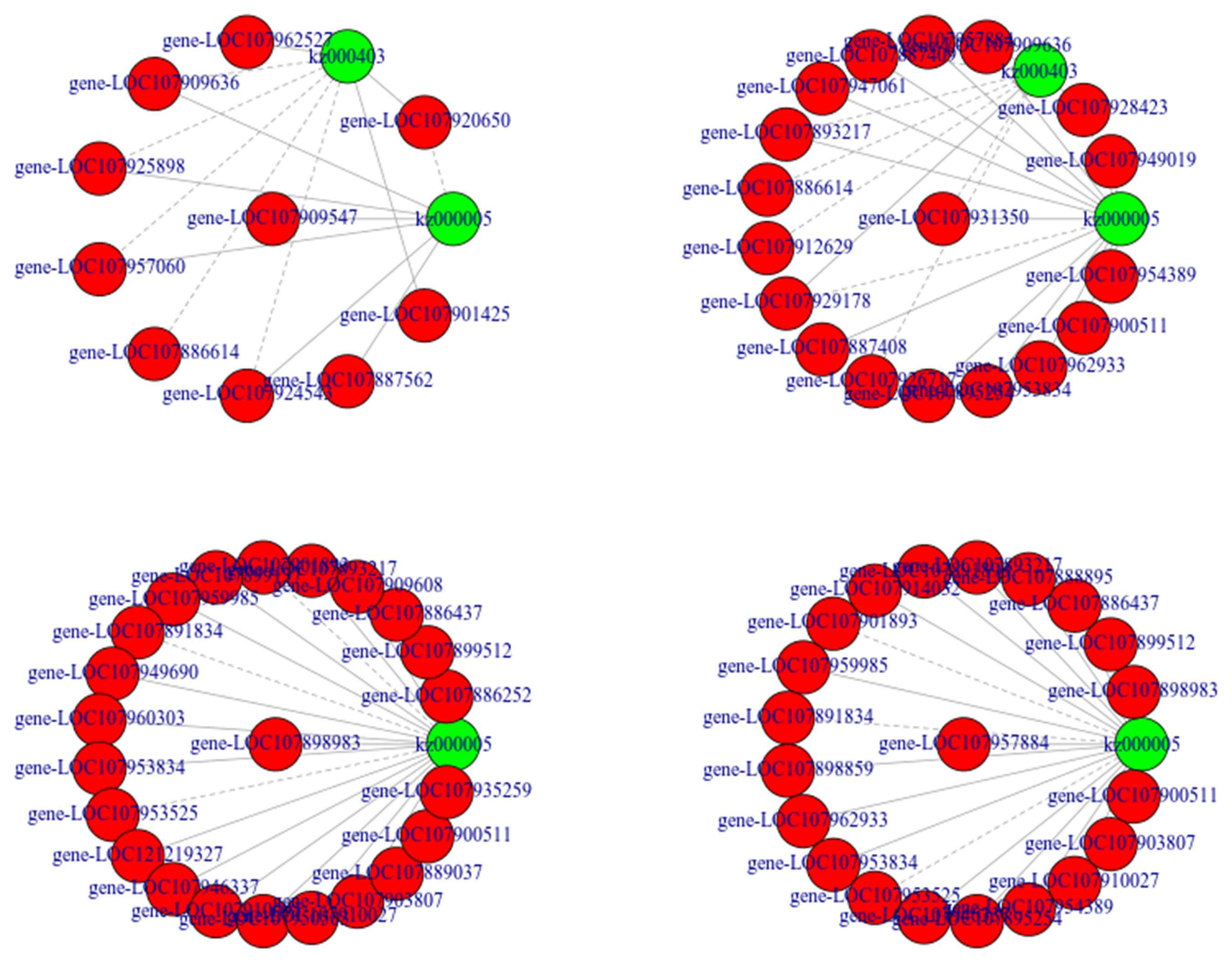

Supplement: Supplementary file 1 [file genes-14-01825-s001.zip › Figure S2-.tif]
